# Supplementary figures and images for: Operation of a brain-computer interface walking simulator for individuals with spinal cord injury
Source: J Neuroeng Rehabil. 2013 Jul 17;10:77. doi: 10.1186/1743-0003-10-77 (PMC3723437; doi:10.1186/1743-0003-10-77)

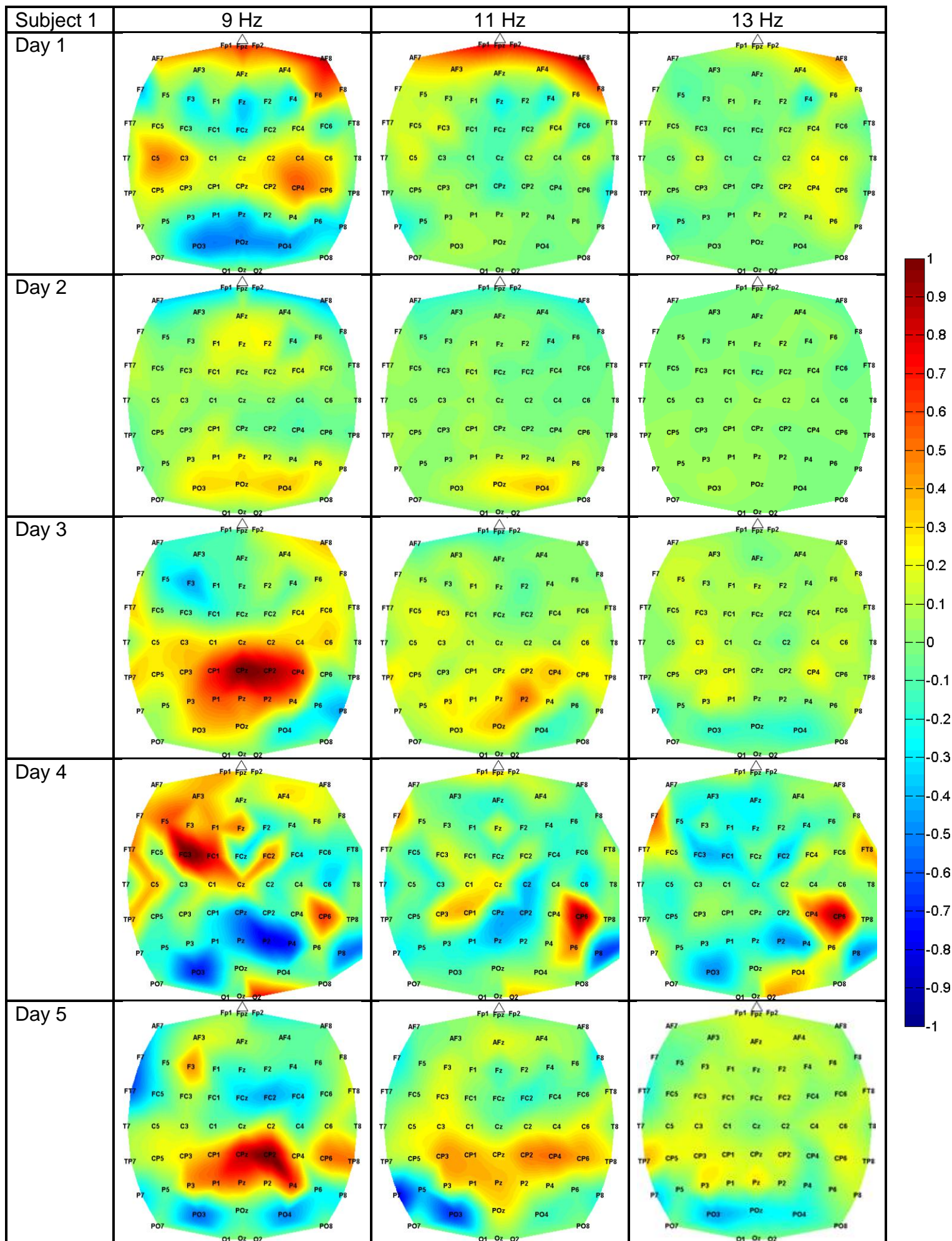

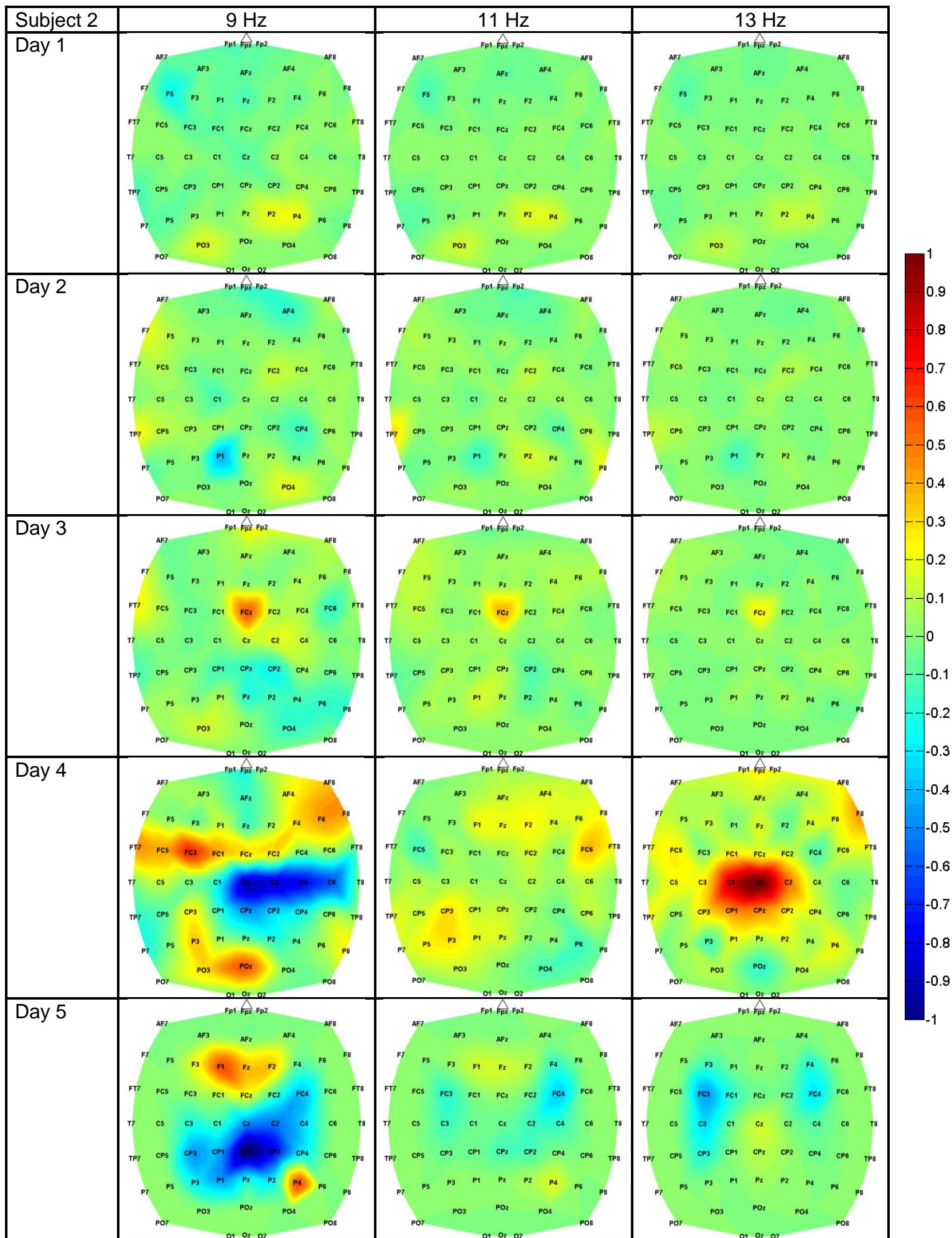

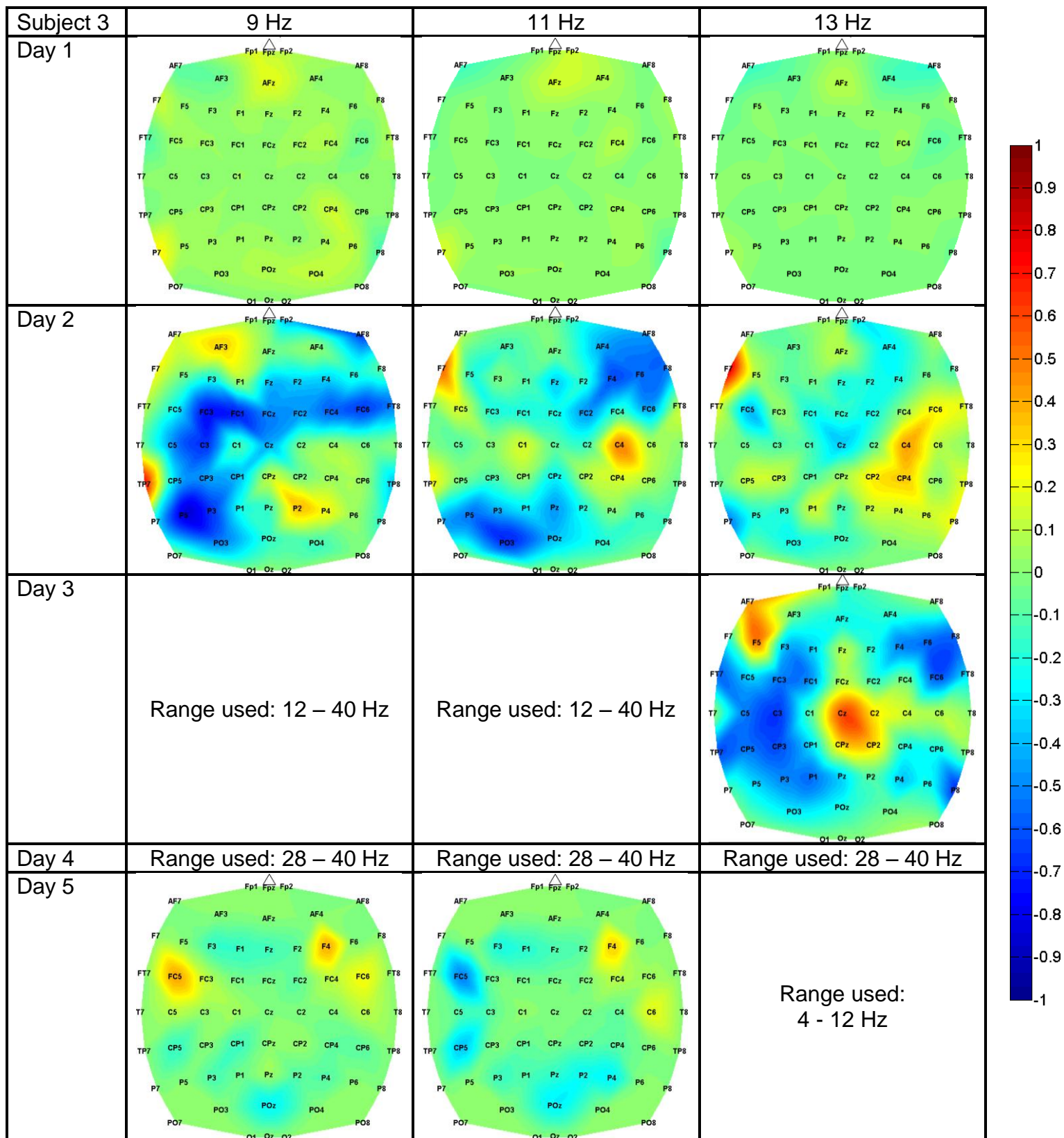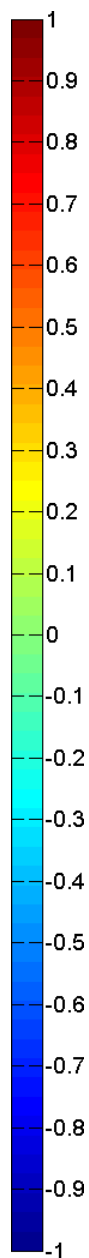

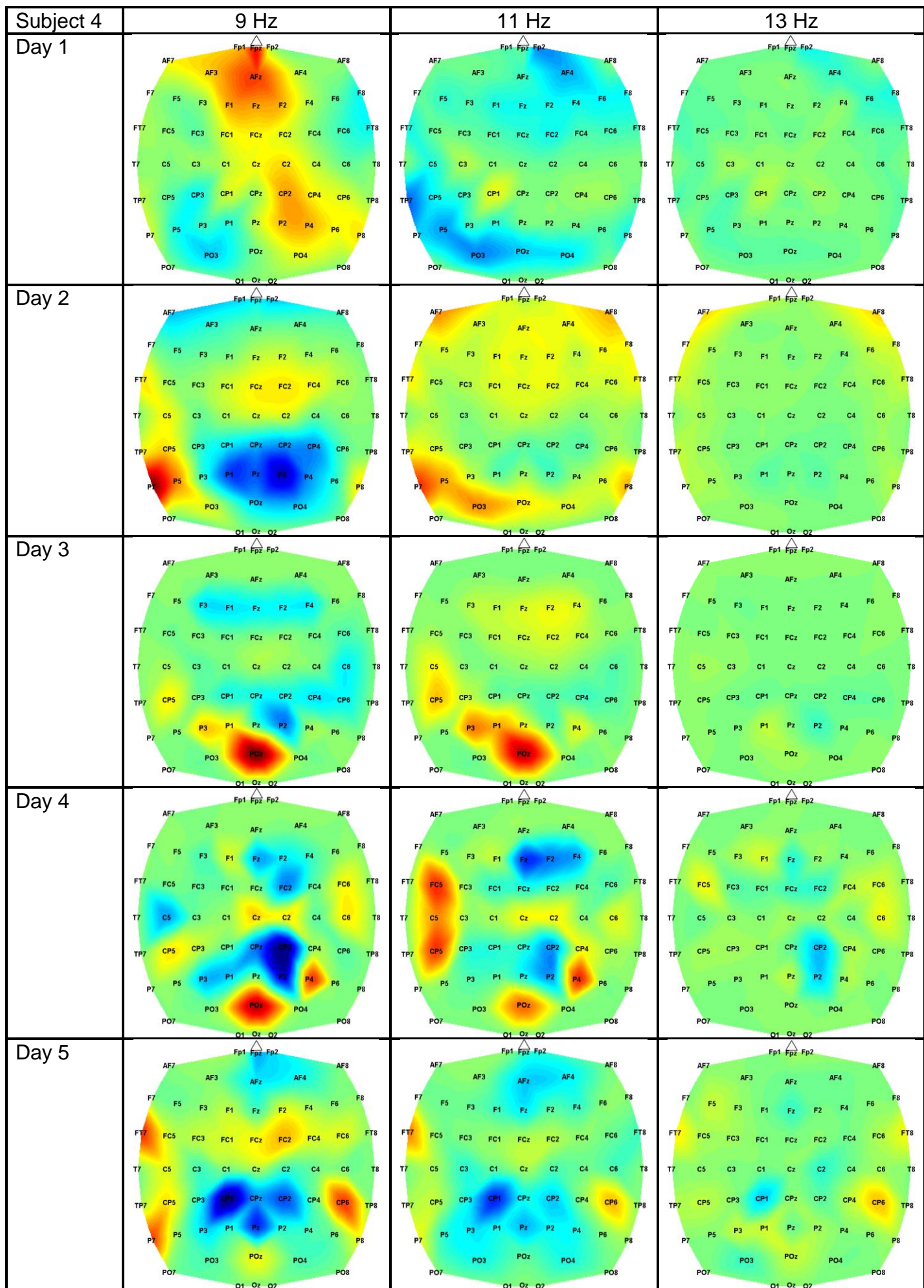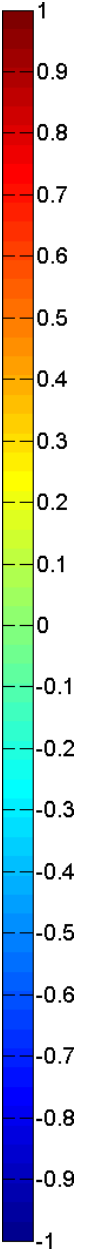

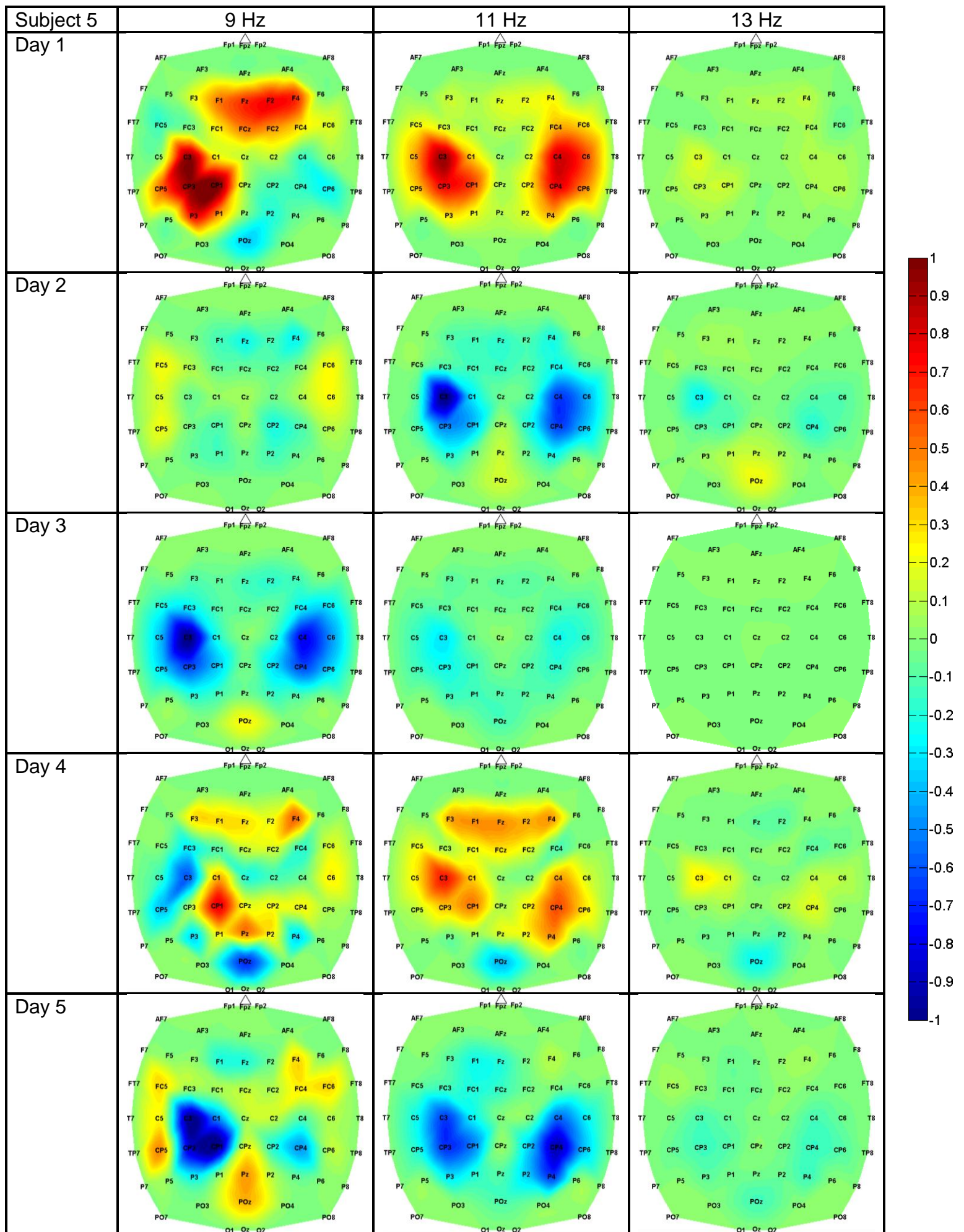

Supplement: Additional file 1 — Feature extraction images of all participants for all experimental sessions over the 8-14 Hz frequency band. Dark colors (red and blue) represent the areas that were responsible for encoding the differences between idling and walking KMI. [file 1743-0003-10-77-S1.pdf]
